# In vitro cellular models of human hepatic fatty acid metabolism: differences between Huh7 and HepG2 cell lines in human and fetal bovine culturing serum

**DOI:** 10.14814/phy2.13532

**Published:** 2017-12-21

**Authors:** Pippa J. Gunn, Charlotte J. Green, Camilla Pramfalk, Leanne Hodson

**Affiliations:** ^1^ Oxford Centre for Diabetes, Endocrinology and Metabolism Radcliffe Department of Medicine University of Oxford Churchill Hospital Oxford United Kingdom; ^2^ Division of Clinical Chemistry Department of Laboratory Medicine Karolinska Institutet at Karolinska University Hospital Huddinge Stockholm Sweden; ^3^ National Institute for Health Research Oxford Biomedical Research Centre Oxford University Hospital Trusts Oxford United Kingdom

**Keywords:** Cell lines, human serum, liver, triacylglycerol

## Abstract

Human primary hepatocytes are the gold standard for investigating lipid metabolism in nonalcoholic fatty liver disease (NAFLD); however, due to limitations including availability and donor variability, the hepatoma cell lines Huh7 and HepG2 are commonly used. Culturing these cell lines in human serum (HS) has been reported to improve functionality; however, direct comparison of fatty acid (FA) metabolism in response to culturing in HS is lacking. The aim of this study was to compare FA metabolism between HepG2 and Huh7 cells in response to culturing in different sera. Both HepG2 and Huh7 cells were grown in media containing 11 mmol/L glucose and either 2% HS or 10% fetal bovine serum. After 3 days, insulin and insulin‐like growth factor‐1 signaling were measured. At 7 days, intracellular triacylglycerol (TAG) and media 3‐hydroxybutyrate, TAG and apolipoprotein B were measured, as was the FA composition of intracellular TAG and phospholipids. Both cell lines demonstrated higher levels of polyunsaturated fatty acid content, increased insulin sensitivity, higher media TAG levels and increased FA oxidation when cultured in HS. Notably, independent of serum type, Huh7 cells had higher intracellular TAG compared to HepG2 cells, which was in part attributable to a higher de novo lipogenesis. Our data demonstrate that intrahepatocellular FA metabolism is different between cell lines and influenced by culturing sera. As a result, when developing a physiologically‐relevant model of FA metabolism that could be developed for the study of NAFLD, consideration of both parameters is required.

## Introduction

Non‐alcoholic fatty liver disease (NAFLD) has become a significant public health burden (Younossi et al. 2016). NAFLD covers a spectrum of conditions, the first of which is steatosis, where lipid droplets (LDs) primarily containing triacylglycerol (TAG), form within hepatocytes. Increased intrahepatocellular storage of TAG can result from elevated delivery or synthesis of fatty acids (FAs), reduced secretion or oxidation of FAs, or both: several of these processes have been shown to be perturbed in individuals with NAFLD (Diraison et al. 2003; Fabbrini et al. 2008; Lambert et al. 2014; Green et al. 2015b).

Studying the metabolic and mechanistic processes involved in the pathogenesis of human NAFLD is difficult due to the models available. Investigating liver metabolism in humans in vivo is challenging and relies on proxy measurements (e.g., use of stable isotope tracers). In situations where liver biopsies are obtained, the amount of tissue available for study may be limited, or a control group for comparison, lacking (Steenbergen et al. 2013; Green et al. 2015a). For in vitro cellular studies primary human hepatocytes are considered the gold standard; however, they present issues with availability, inter‐donor variability and the short time frame during which they remain differentiated (Steenbergen et al. 2013; Green et al. 2015a). As a result, proliferating human hepatoma cell models are the most widely used option.

Two well‐established cell lines are HepG2 and Huh7; however, these models are poorly differentiated, often resemble fetal liver cells and have limited ability to carry out metabolic processes including TAG hydrolysis and very low‐density lipoprotein (VLDL) secretion (Javitt 1990; Gibbons et al. 1994; Wu et al. 1996), which are key to studying NAFLD pathophysiology. Efforts to overcome these limitations show that both cell lines demonstrate changes in their metabolic phenotype when cultured in human serum (HS), including improvements in expression of lipid metabolism‐regulating nuclear receptors and lipoprotein and bile acid levels (Pramfalk et al. 2016; Steenbergen et al. 2013).

Although there are many reported similarities in the function of HepG2 and Huh7 cells, studies directly comparing the two cell lines are surprisingly sparse (Meex et al. 2011; Green et al. 2015b). As a result, the aim of this work was to comprehensively characterize FA metabolism in both HepG2 and Huh7 cells and investigate whether human serum could improve their functionality.

## Material and Methods

### Materials

All reagents were obtained from Life Technologies (Paisley, UK) unless otherwise stated. Fetal bovine serum (FBS) and HS were purchased from Seralab (Haywards Heath, UK). DC protein assay, TGX gels and midi transfer packs were all purchased from Bio‐Rad (Hemel Hempstead, UK). BLUeye Prestained Protein Ladder was purchased from Geneflow (Lichfield, UK). LONG^®^ R3 insulin‐like growth factor (IGF)‐1 (human) was purchased from Sigma‐Aldrich (Gillingham, UK). Anti‐pan Akt and anti‐p‐Akt (serine 473) were purchased from Cell Signaling Technologies (MA, USA), anti‐*β*‐actin from Abcam (Cambridge, UK) and anti‐apoB from Santa Cruz Biotechnology (CA, USA). Amicon Ultra Centrifugal Filter units were purchased from Millipore (Watford, UK). RNEasy^®^ Plus mini kit was purchased from QIAGEN Sciences (Manchester, UK) and KAPA PROBE FAST qPCR MasterMix (2X) from Kapa Biosystems (London, UK). Taqman probes were purchased from Applied Biosystems (Hemel Hempstead, UK). TAG, lactate and 3‐hydroxybutyrate (3‐OHB) assays were from Instrumentation Laboratory UK (Cheshire, UK). Heavy water (^2^H_2_O) was purchased from CK Isotopes (Ibstock, UK) and gas chromatography (GC) standards were from Sigma‐Aldrich (Gillingham, UK).

### Cell culture

Human hepatoma cells were a gift from Dr Karl Morten (HepG2; University of Oxford) and Dr Camilla Pramfalk (Huh7; Karolinska Institutet, originally from Creative Dynamics Inc. NY, US). Cells were maintained at 37°C in 5% carbon dioxide in low‐glucose (5.5 mmol/L) Dulbecco's modified eagle's medium (DMEM) + Glutamax™ with 10% FBS, 1% nonessential amino acids (NEAAs) and 10,000 U/mL penicillin‐streptomycin.

### Experimental treatments

After passaging, cells were seeded and grown to confluence in maintenance media (as described above). Upon reaching confluence they were washed twice with PBS and treatment media was added. Treatment media consisted of phenol red‐free DMEM, 11 mmol/L glucose, 1% NEAAs, 10,000 U/mL penicillin‐streptomycin, sodium pyruvate and Glutamax™, with 10% FBS or 2% HS. For assessment of de novo lipogenesis (DNL), 10% heavy water (^2^H_2_O) was added to media and for all experiments, media was changed every 48–72 h.

Data were initially analyzed at three, seven, and 14 days, but since longer culture time did not affect RNA expression, intracellular or media TAG and some transcription factors demonstrated a change in expression from day seven, only the 7 day results are presented. In addition, cells were cultured in 2% FBS to ensure differences between FBS and HS were not due to serum content of media; however, cell death occurred in Huh7 cells after 3 days and this condition was, therefore, not pursued further.

Fully confluent Huh7 or HepG2 cells were cultured for 72 h in either 10% FBS or 2% HS before serum starvation for 2 h. During the penultimate 10 min of serum starvation cells were stimulated with either insulin or IGF‐1 (333, 111, 37, 12.3, 4.1, 1.4, 0.46 or 0 nmol/L). Lysates were then collected and stored at −80°C until analyzed.

Protein concentrations were determined using the DC™ protein assay. Briefly, 20 *μ*g of whole cell lysates were subjected to SDS‐PAGE using Bio‐Rad TGX stain free precast gels (4–20%) and transferred to polyvinylidene difluoride (PVDF) membranes using Bio‐Rad Trans‐blot^®^ turboTM transfer system. PVDF membranes were probed with primary antibodies raised against the protein of interest, as indicated in the figure legends. Detection of primary antibodies was performed using appropriate peroxidase‐conjugated IgG, and protein signals were visualized using enhanced chemiluminescence and ChemiDoc imaging system. Quantification of immunoblots was done using Image J software (NIH, Bethesda, MD; http://rsb.info.nih.gov/ij). For apoB, cell media was concentrated using Amicon Ultra Centrifugal Filter units as described previously (Green et al. 2015a). Medium that had not been added to cells was treated in the same way as experimental samples as a background control.

Phosphorylation of Akt (serine 473) was measured by ELISA according to manufacturer's instructions. Briefly, cell lysates were diluted 1/10 and bound to plates for 2 h before a detector antibody (Akt serine 473) was added for 1 h, and finally a horseradish peroxidase secondary antibody was added for 30 min, all at room temperature. Stabilized chromogen was then added for 30 min and the reaction was terminated by the addition of a stop solution. Absorbance was measured at 450 nm on a VERSAmax Microplate Reader. Akt phosphorylation was quantified from a standard curve and normalized to protein concentration.

RNA was extracted using the RNEasy^®^ Plus mini kit and cDNA was synthesized using 1 *μ*g RNA with the High‐Capacity cDNA Reverse Transcription Kit, both according to manufacturer's instructions. Reverse transcription was carried out on an MJ Research Tetrad PTC‐225 Thermal Cycler (GMI, Inc; MN, USA) with annealing at 25°C for 10 min and cDNA synthesis at 37°C for 120 min. Termination of the reaction occurred after 5 min at 85°C.

Quantitative PCR was carried out on an ABI PRISM^®^ 7900HT Sequence Detection System (Applied Biosystems; Warrington, UK). For each gene, samples were diluted 1:40 and measured in triplicate using a TaqMan^®^ probe labeled with FAM and 2X KAPA PROBE FAST Master Mix. The reaction was held at 95°C for 3 min, followed by 40 cycles of denaturation and annealing for 3 sec at 95°C and for 20 sec 60°C, respectively. A standard curve consisting of pooled cDNA from all samples was included for each gene, along with a no‐template and no‐enzyme control.

Ct values generated were used to calculate a relative expression ratio as described previously (Pfaffl 2001). Each sample was expressed relative to a calibrator sample and corrected to the geometric mean of three reference genes (*B2M*,* HPRT1*, and *YWHAZ*).

### Intracellular TAG and media TAG, lactate and 3‐OHB concentrations

TAG, lactate and 3‐OHB were measured in collected media. For intracellular TAG, cells were lysed and heated to 95°C before being centrifuged at 15,000*g* for 10 min and supernatant removed for analysis on the ILab 650 (Instrumentation Laboratory, Werfen; Warrington, UK).

All assays were carried out according to manufacturer's instructions and used an appropriate calibrator or standard curve for quantification, as well as quality control samples. Assays were previously optimized for low concentrations found in vitro (Green et al. 2015a), with complete media used for a background measurement and results normalized to protein concentration.

### Lipid extraction and gas chromatography‐mass spectrometry

Lipid was extracted from cell lysate and media according to the Folch et al. (1957) method. Internal standards containing known concentrations of glyceryl triheptadecanoate (15:0) and 1,2‐Diheptanoyl‐sn‐glycero‐3‐phosphocholine (17:0) were added to samples and fatty acid methyl esters (FAMEs) from TAG and phospholipids (PLs) were prepared and analyzed by a 6890N Network GC System (Agilent Technologies; CA, USA) as described previously (Burdge et al. 2000). FAMEs were identified by their retention times compared to a standard containing 31 known FAs and quantified in micromolar from the peak area based on their molecular weight. The micromolar quantities were then totaled and each fatty acid was expressed as a percentage of this value (molar percentage; mol%).

Intracellular DNL was assessed based on the incorporation of deuterium from ^2^H_2_O in the media (Finnigan GasBench‐II, ThermoFisher Scientific, UK) into [^2^H]palmitate in intracellular TAG and PL and media TAG. [^2^H]palmitate enrichments were determined simultaneously by GC‐mass spectrometry (GC–MS) using a 5890 GC coupled to a 5973N MSD (Agilent Technologies; CA, USA). Ions with mass‐to‐charge ratios (*m*/*z*) of 270 (M + 0) and 271 (*M* + 1) were determined by selected ion monitoring (Semple et al. 2009). For simplicity, ‘DNL’ refers to the proportion of newly synthesized palmitate in intracellular TAG and PL and media TAG and represents the synthesis of fatty acids from nonlipid precursors.

### Statistics

All experiments consisted of a minimum of three replicates and data are presented as mean ± SD. GraphPad Prism 7 (GraphPad Software, Inc.; La Jolla, CA) was used for data analysis. One‐ and two‐way ANOVAs with Bonferroni's post hoc multiple comparison tests were used to determine significant differences (*P *<* *0.05).

## Results

### Storage and synthesis of TAG is higher in Huh7 cells than HepG2 cells in response to HS and FBS

**Figure 1:**
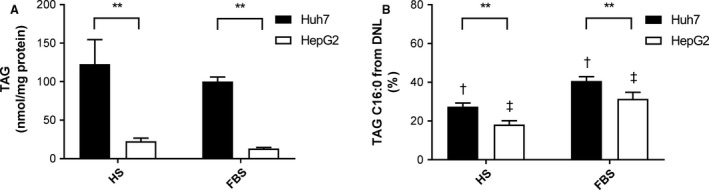
Storage and synthesis of triacylglycerol (TAG) in Huh7 and HepG2 cells. Cells were cultured in either 2% human serum (HS) or 10% fetal bovine serum (FBS)‐containing media for seven days. (A) Intracellular TAG content (*n *=* *5) and (B) palmitate (C16:0) in TAG derived from de novo lipogenesis (DNL;* n *=* *3)) were measured. Data are mean ± SD. *P <* *0.05, **P <* *0.001; matching symbols denote significant differences between means for Huh7 (^†^) and HepG2 cells (^‡^), *P *<* *0.05; two‐way ANOVA with Bonferroni's multiple comparisons test.

The primary characteristic of steatosis is a net retention of intrahepatocellular TAG in LDs. Levels of intracellular TAG were found to be significantly higher in Huh7 than HepG2 cells when cultured in both sera (Fig. 1A). Also independent of culturing serum, Huh7 cells had a significantly higher proportion of DNL‐derived palmitate than HepG2 cells; for both cell lines DNL was higher when cultured in FBS compared to HS (*P *<* *0.05, Fig. 1B).

In contrast, gene expression analysis showed an upregulation of DNL‐related genes (acetyl‐CoA carboxylase 1; *ACACA* and fatty acid synthase; *FASN*) in HepG2 compared to Huh7 cells (Table 1). This may be due to a five to 14‐fold higher expression of the lipogenic transcription factor carbohydrate‐responsive element binding protein (ChREBP; *MLXIPL*) in HepG2 cells, compared to a more modest elevation of sterol‐regulatory element binding protein (SREBP)‐1c (*SREBF1*) expression in Huh7 cells (all *P *<* *0.05, Table 1). Genes involved in lipid transport (liver fatty acid binding protein 1; *FABP1*) and storage (cell death inducing DFFA like effector C; *CIDEC)* were also expressed more highly in Huh7 compared to HepG2 cells (*P *<* *0.05).

**Table 1:** mRNA expression of lipid metabolism genes in Huh7 and HepG2 cells cultured with 2% HS or 10% FBS

	HS		FBS	
	Huh7	HepG2	Huh7	HepG2
FA transport and synthesis				
*ACACA*	0.67 ± 0.05	1.27 ± 0.12**	0.74 ± 0.08	1.32 ± 0.10**
*ACACB*	0.96 ± 0.16	1.60 ± 0.50* ^,^ ‡	0.86 ± 0.05	1.00 ± 0.11‡
*CIDEC*	1.40 ± 0.30†	0.32 ± 0.05**	0.77 ± 0.19†	0.23 ± <0.01*
*DGAT2*	1.52 ± 0.34	1.04 ± 0.06	1.47 ± 0.43	0.66 ± 0.15*
*FASN*	0.60 ± 0.06	1.01 ± 0.13*, ‡	0.66 ± 0.08	1.35 ± 0.23** ^,^ ‡
*FABP1*	1.44 ± 0.31	0.71 ± 0.05*	1.37 ± 0.12	0.68 ± 0.08*
*SCD1*	0.98 ± 0.21	1.30 ± 0.09	1.03 ± 0.22	1.40 ± 0.28
FA elongation and desaturation				
*ELOVL2*	0.77 ± 0.06	1.75 ± <0.01** ^,^ ‡	0.85 ± 0.10	1.15 ± 0.15*, ‡
*ELOVL5*	0.93 ± 0.14	1.07 ± 0.02	1.03 ± 0.11	1.22 ± 0.05
*ELOVL6*	1.27 ± 0.17	0.80 ± 0.12*	1.56 ± 0.19	0.85 ± 0.08**
*FADS1*	1.10 ± 0.22	0.60 ± 0.07*	1.22 ± 0.27	0.59 ± 0.10*
*FADS2*	1.45 ± 0.18	0.51 ± 0.05**	1.58 ± 0.24	0.51 ± 0.09**
Lipolysis, secretion, and oxidation				
*APOB*	0.65 ± 0.07	1.70 ± 0.26** ^,^ ‡	0.60 ± 0.12	0.93 ± 0.05* ^,^ ‡
*CES1*	1.45 ± 0.14†	0.01 ± <0.01**	2.25 ± 0.19	0.01 ± <0.01**
*CIDEB*	1.14 ± 0.21	0.21 ± 0.02**	0.98 ± 0.26	0.20 ± 0.08*
*CPT1A*	1.22 ± 0.25	0.59 ± 0.07*	1.37 ± 0.24	0.66 ± 0.05**
*LIPC*	1.34 ± 0.28	0.32 ± 0.06**	1.03 ± 0.35	0.18 ± 0.04*
*MTTP*	1.63 ± 0.40	0.74 ± 0.01*	1.47 ± 0.10	0.39 ± 0.04**
*PNPLA2*	0.52 ± 0.09	1.80 ± 0.28** ^,^ ‡	0.54 ± 0.08	1.12 ± 0.15** ^,^ ‡
*PNPLA3*	1.26 ± 0.10	0.92 ± 0.12**	1.17 ± 0.10	0.54 ± 0.04*
*TM6SF2*	1.57 ± 0.07	0.55 ± 0.10**	1.19 ± 0.17	0.41 ± 0.05*
Hepatic nuclear receptors				
*HNF4A*	1.25 ± 0.21	1.11 ± 0.12	1.22 ± 0.21	0.83 ± 0.04*
*NR1H3*	1.33 ± 0.08	0.99 ± 0.22*	1.21 ± 0.23	0.70 ± 0.22**
*NR1H4*	1.29 ± 0.22	0.55 ± 0.07	1.30 ± 0.34	0.19 ± 0.08*
Transcription factors				
*MLXIPL*	0.19 ± 0.06	2.68 ± 0.97** ^,^ ‡	0.19 ± 0.03	1.11 ± 0.14* ^,^ ‡
*PPARA*	1.19 ± 0.14	0.76 ± 0.11*	1.03 ± 0.19	0.64 ± 0.10*
*PPARG*	1.08 ± 0.26	0.48 ± 0.22*	1.03 ± 0.23	0.28 ± 0.06*
*PPARGC1A*	0.02 ± <0.01	2.78 ± 0.27**	0.01 ± <0.01	2.46 ± 0.54**
*SREBF1*	0.92 ± 0.05	0.62 ± 0.04*	0.83 ± 0.15	0.75 ± 0.04

Cells were cultured in either 2% human serum (HS) or 10% fetal bovine serum (FBS)‐containing media for seven days. mRNA expression is presented relative to a calibrator sample, all genes were corrected to three reference genes (*B2M*,* HPRT1*, and *YWHAZ*). Data are mean ± SD, *n *=* *4. Two‐way ANOVA with Bonferroni's post hoc tests. *P <* *0.05, **P <* *0.001 versus Huh7 cells in the same serum condition; matching symbols denote significant differences between means for Huh7 (^†^) and HepG2 cells (^‡^), *P *<* *0.05.

### Composition of FAs in TAG and PL fractions differ between cell lines and culturing sera

**Figure 2:**
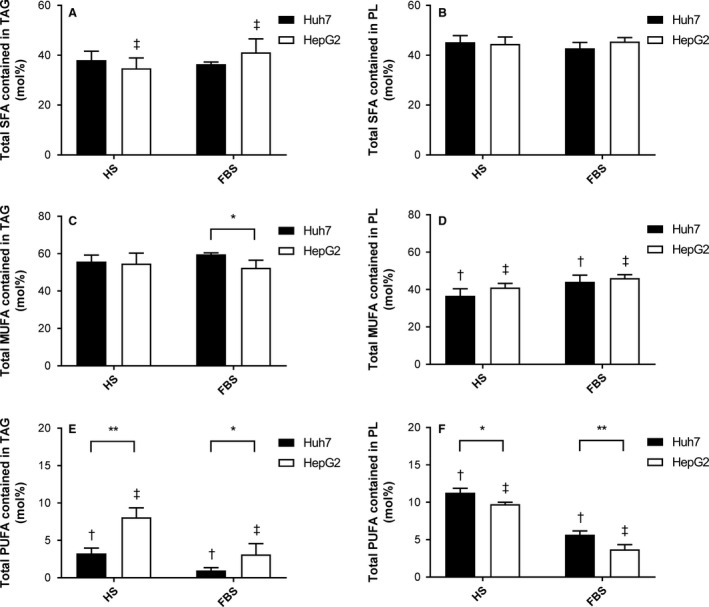
Metabolism and composition of triacylglycerol (TAG) and phospholipid (PL) fractions in Huh7 and HepG2 cells. Cells were cultured in either 2% human serum (HS) or 10% fetal bovine serum (FBS)‐containing media for seven days. Intracellular TAG was extracted and the contribution of saturated, monounsaturated and polyunsaturated fatty acids (SFA, MUFA and PUFA) was determined for TAG (A, C, E) and PL (B, D, F). Data are mean ± SD,* n *=* *5. *P <* *0.05, **P <* *0.001; matching symbols denote significant differences between means for Huh7 (^†^) and HepG2 cells (^‡^), *P *<* *0.05; two‐way ANOVA with Bonferroni's multiple comparisons test.

Given the differences observed in TAG synthesis and accumulation, the FA composition of TAG and PLs were measured. Total saturated fatty acids (SFAs) in the both lipid fractions were similar between cell lines and sera (Fig. 2A and B); although Huh7 cells had a greater proportion of C18:0 (stearate) in the PL fraction while HepG2 cells had more C14:0 (myristate) and C16:0 (palmitate; all *P *<* *0.05, Table 3). Total intracellular monounsaturated FA (MUFA) composition showed an effect of serum on the PL fraction, with higher levels in FBS‐cultured compared to HS‐cultured cells (*P *<* *0.05, Fig. 2C and D). Inter‐cell differences were apparent in individual FA composition, with significantly higher levels of C16:1*n*‐7 (palmitoleate) and C18:1*n*‐9 (oleate) and lower levels of C18:1*n*‐7 (vaccinate) in Huh7 compared to HepG2 cells in both TAG and PL fractions (Tables 2 and 3).

**Table 2:** Fatty acid composition of triacylglycerol in 2% Huh7 and 10% HepG2 cells cultured with HS or FBS**.**

	HS		FBS	
	Huh7	HepG2	Huh7	HepG2
Saturated fatty acids				
14:0	5.90 ± 0.56	4.72 ± 0.35	4.22 ± 0.21	6.25 ± 1.03
16:0	27.0 ± 2.40	26.1 ± 2.90	24.8 ± 0.8	29.1 ± 3.5*
18:0	5.17 ± 0.63†	3.95 ± 1.23‡	7.44 ± 0.90†	5.84 ± 1.06* ^,^ ‡
Monounsaturated fatty acids				
16:1*n*‐7	15.2 ± 1.00	8.3 ± 1.3** ^,^ ‡	14.0 ± 1.0	11.8 ± 1.00* ^,^ ‡
18:1*n*‐9	30.1 ± 1.9†	25.6 ± 1.4** ^,^ ‡	35.6 ± 0.9†	22.9 ± 2.0** ^,^ ‡
18:1*n*‐7	10.5 ± 1.10	20.8 ± 3.9**	10.1 ± 0.5	17.7 ± 3.0**
Polyunsaturated fatty acids				
18:2*n*‐6	2.29 ± 0.37†	4.71 ± 0.73** ^,^ ‡	0.30 ± 0.03†	0.78 ± 0.29‡
18:3*n*‐6	0.19 ± 0.07	0.44 ± 0.21	0.31 ± 0.21	0.54 ± 0.18
18:3*n*‐3	0.14 ± 0.10†	0.39 ± 0.15* ^,^ ‡	0.00 ± 0.00†	0.00 ± 0.00‡
20:4*n*‐6	0.18 ± 0.09	0.66 ± 0.12*	0.09 ± 0.05	0.72 ± 0.44*
20:5*n*‐3	0.47 ± 0.26	1.97 ± 1.05*	0.29 ± 0.13	1.08 ± 0.61

Cells were cultured in either 2% human serum (HS) or 10% fetal bovine serum (FBS)‐containing media for seven days. Data are mean ± SD, *n *=* *5. Two‐way ANOVA with Bonferroni's post hoc tests. *P <* *0.05, **P <* *0.001 versus Huh7 cells in the same serum condition; matching symbols denote significant differences between means for Huh7 (^†^) and HepG2 cells (^‡^), *P *<* *0.05.

**Table 3:** Fatty acid composition of phospholipids in Huh7 and HepG2 cells cultured with 2% HS or 10% FBS

	HS		FBS	
	Huh7	HepG2	Huh7	HepG2
Saturated fatty acids				
14:0	0.90 ± 0.10	3.76 ± 0.69** ^,^ ‡	0.83 ± 0.23	6.38 ± 1.74** ^,^ ‡
16:0	31.2 ± 1.50	35.8 ± 2.1*	30.5 ± 1.50	33.4 ± 1.6*
18:0	13.1 ± 1.30	5.1 ± 0.4**	11.5 ± 1.20	5.7 ± 0.9**
Monounsaturated fatty acids				
16:1*n*‐7	8.50 ± 1.86	9.75 ± 1.62	9.20 ± 2.03	13.85 ± 4.58*
18:1*n*‐9	20.9 ± 1.2†	15.9 ± 1.4** ^,^ ‡	29.2 ± 2.4†	19.0 ± 1.3** ^,^ ‡
18:1*n*‐7	7.0 ± 1.5	14.9 ± 3.4**	5.4 ± 0.3	12.5 ± 3.3**
20:1*n*‐9	0.29 ± 0.14	0.57 ± 0.07*	0.29 ± 0.14	0.73 ± 0.06**
Polyunsaturated fatty acids				
18:2*n*‐6	5.16 ± 0.16†	5.58 ± 0.56‡	0.49 ± 0.05†	0.69 ± 0.12‡
18:3*n*‐6	0.47 ± 0.07†	0.26 ± 0.02** ^,^ ‡	0.19 ± 0.03†	0.18 ± 0.05‡
18:3*n*‐3	0.07 ± 0.02†	00.1 ± <0.01	0.16 ± 0.01†	0.09 ± 0.04**
20:4*n*‐6	2.79 ± 0.61†	1.51 ± 0.26**	1.70 ± 0.19†	0.97 ± 0.04*
20:5*n*‐3	0.38 ± 0.05†	0.48 ± 0.14‡	0.61 ± 0.04†	0.31 ± 0.12** ^,^ ‡
22:5*n*‐3	1.22 ± 0.29	0.57 ± 0.05**	1.49 ± 0.25	0.92 ± 0.25*
22:6*n*‐3	1.21 ± 0.48	1.77 ± 0.36* ^,^ ‡	1.04 ± 0.06	0.84 ± 0.12‡

Cells were cultured in either 2% human serum (HS) or 10% fetal bovine serum (FBS)‐containing media for 7 days. Data are mean ± SD, *n *=* *5. Two‐way ANOVA with Bonferroni's post hoc tests. *P <* *0.05, **P <* *0.001 versus Huh7 cells in the same serum condition; matching symbols denote significant differences between means for Huh7 (^†^) and HepG2 cells (^‡^), *P *<* *0.05.

Differences in polyunsaturated FA (PUFA) content were more marked between the cell lines for both TAG and PL fractions (Fig. 2E and F). Notably, the two essential FAs, C18:2*n*‐6 (linoleate) and C18:3*n*‐3 (*α*‐linolenate), were significantly higher in abundance in the TAG and PL fractions as a result of culturing in HS rather than FBS (Fig. 2E and F, Table 2 and 3). However, partitioning of these FAs was dependent on cell line: in the TAG fraction, PUFAs were higher in HepG2 cells (*P *<* *0.05, Fig. 2E), while Huh7 cells had a significantly higher abundance of PUFA in the PL fraction compared to HepG2 cells (Fig. 2F). This was reflected in all individual PUFAs except C18:2*n*‐6 (Table 3), suggesting that Huh7 cells partition PUFAs into PL rather than store them in the TAG fraction.

Differences in FA composition can result from intracellular metabolism or exogenous supply. As essential FAs cannot be synthesized de novo, the increase in intracellular content can be attributed to higher levels contained in HS; this was confirmed when serum alone was analyzed. Media with 2% HS contained twice as much TAG compared to media with 10% FBS, including 10 times the amount of C18:2*n*‐6 and a small amount of C18:3*n*‐3, which was undetectable in FBS. However, longer chain *n*‐3 FAs were not detected in either serum, demonstrating the ability of both cell lines to elongate and desaturate FAs, although potentially to different degrees. Expression of FA desaturases (*FADS1* and *FADS2*) were expressed more highly in Huh7 cells, regardless of culturing serum, but elongases were differentially upregulated in Huh7 cells (*ELOVL6*) and HepG2 cells (*ELOVL2*; all *P *<* *0.05, Table 1).

### TAG and FA disposal is upregulated by HS but differs between cell lines

**Figure 3:**
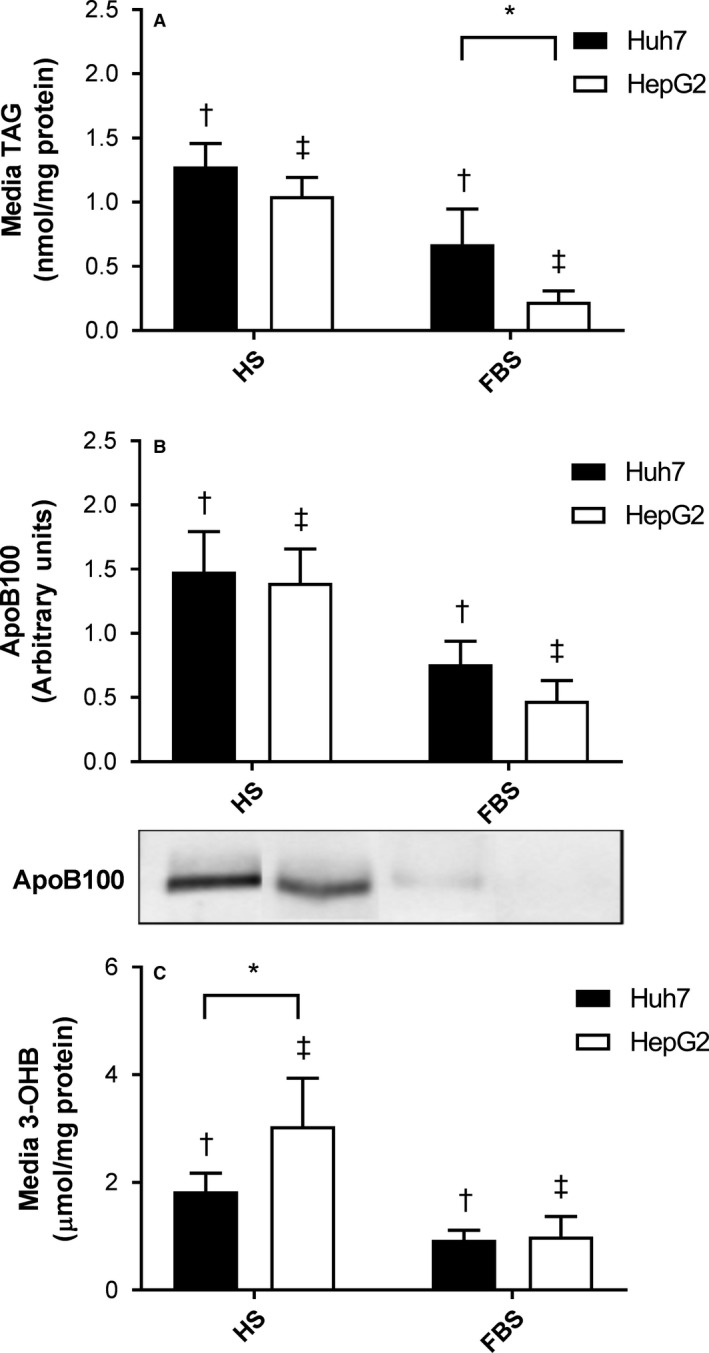
Secretion and oxidation of triacylglycerol (TAG) and fatty acid oxidation in Huh7 and HepG2 cells. Cells were cultured in either 2% human serum (HS) or 10% fetal bovine serum (FBS)‐containing media for 7 days. (A) TAG (*n *=* *5), (B) apolipoprotein (apo)B100 (*n *=* *4), and (C) 3‐hydroxybutyrate (3‐OHB;* n *=* *5) in cell media were measured. Data are mean ± SD. * *P *<* *0.05, **P <* *0.001; matching symbols denote significant differences between means for Huh7 (^†^) and HepG2 cells (^‡^), *P *<* *0.05; two‐way ANOVA with Bonferroni's multiple comparisons test.

The ability of hepatocytes to mobilize TAG for secretion in lipoproteins and FAs for oxidation is an important part of their metabolic function, and is often lacking in hepatoma cell lines. We assessed the cell media TAG concentration as a proxy marker of TAG secretion and found it to be significantly higher in Huh7 compared to HepG2 cells in FBS (Fig. 3A). In both cell lines media TAG was significantly higher when cultured in HS compared to FBS, but the levels did not significantly differ between Huh7 and HepG2 cells after culturing in HS (Fig. 3A). ApoB100, the apolipoprotein associated with particles secreted from hepatocytes, was significantly more abundant in the media after culturing in HS than FBS, but did not differ between cell lines (Fig. 3B). In contrast, levels of *APOB* mRNA were only elevated after culturing in HS in HepG2 cells (*P *<* *0.05), and were significantly higher in HepG2 cells than Huh7 cells in both conditions. Expression of *MTTP*,* CIDEB* and *TM6SF2*, genes related to the transfer of TAG to apoB100 containing particles and their secretion, were significantly higher in Huh7 cells in both sera (Table 1).

As a marker of FA oxidation, 3‐OHB in the media was measured and was significantly higher in both cells lines after culturing in HS compared to FBS. Media 3‐OHB concentrations were also significantly higher in HepG2 compared to Huh7 cells when cultured in HS (Fig. 3C). In contrast, the expression of oxidative genes (peroxisome proliferator‐activated receptor‐*γ*;* PPARG* and carnitine palmitoyltransferase 1A; *CPT1A*) were more highly expressed in Huh7 cells (*P *<* *0.05), but were unchanged in response to culture serum (Table 1). In line with the ability of both cells to mobilize TAG for secretion and oxidation, expression of lipases were present, although *LIPC, PNPLA3* and *CES1* were all as expressed more highly in Huh7 cells and only *PNPLA2* in HepG2 cells (all *P *<* *0.05, Table 2).

### HS increases insulin sensitivity in both cell lines and suppresses IGF‐1 signaling in Huh7 cells

**Figure 4:**
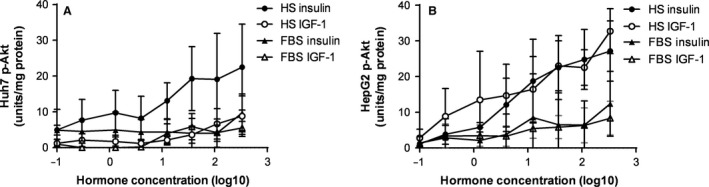
Insulin and insulin‐like growth factor 1 (IGF‐1) signalling in Huh7 and HepG2 cells. Cells were cultured in either 2% human serum (HS) or 10% fetal bovine serum (FBS)‐containing media for 3 days before serum starvation and stimulation with insulin or IGF‐1 at 0, 0.46, 1.4, 4.1, 12.3, 37, 111, and 333 nmol/L. Insulin/IGF‐1 stimulated Akt serine 473 phosphorylation was measured by ELISA in Huh7 (**A**) and HepG2 cells (**B**) Data are mean ± SD,* n *=* *3.

Given the role of insulin in regulating FA metabolism, phosphorylation of Akt in response to insulin and IGF‐1 stimulation was measured. While healthy hepatocytes do not express the IGF‐1 receptor, due to de‐differentiation, hepatoma cell lines do express the receptor and respond to IGF‐1 by phosphorylating Akt in the same way as insulin. HS significantly increased Akt phosphorylation in response to insulin to the same extent in both Huh7 and HepG2 cells (Huh7 AUC: 44.9 ± 7.6 vs. 16.0 ± 3.0, HepG2 AUC: 49.6 ± 6.9 vs. 18.2 ± 6.7; both *P *<* *0.05, Fig. 4A and B). However, in HepG2 cells, IGF‐1 also phosphorylated Akt at a similar magnitude to insulin in HS compared to FBS, which was not seen in Huh7 cells (Huh7 AUC: 11.0 ± 2.4 vs. 8.3 ± 1.8; ns, HepG2 AUC: 57.8 ± 8.4 vs. 16.1 ± 3.2; *P *<* *0.001, Fig. 4A and B). These results were confirmed by immunoblotting of the 111 nmol/L samples (data not shown) and indicate a suppression of perturbed IGF‐1 signaling in Huh7 cells cultured in HS.

## Discussion

The aim of this study was to characterize FA metabolism in HepG2 and Huh7 in response to HS and FBS, in order determine the cell line and culture conditions most suitable for use to develop as an in vitro model of NAFLD. Overall, our results showed several functional changes in response to culturing in 2% HS compared to 10% FBS in both Huh7 and HepG2 cells; DNL decreased, while oxidation and levels of TAG in the media increased. Differences between cell lines were also apparent; Huh7 cells stored and synthesized more TAG and contained more PUFA in the PL fraction, whereas HepG2 cells had a higher abundance of PUFA in the TAG fraction.

We found that Huh7 cells stored more TAG than HepG2 cells in both culturing conditions, which may in part be explained by higher DNL in Huh7 cells. Although Steenbergen et al. (2013) did not measure intracellular TAG, they reported an increase in LDs in Huh7 cells after 21 days of culture in HS; this was attributed to elevated liver X receptor (LXR; *NR1H3*), peroxisome proliferator‐activated receptor (PPAR)‐*α* and PPAR‐*γ* expression. We did not observe changes in these transcripts or an increase in intracellular TAG in response to HS in either cell line, which may be due to the difference in culturing time. However, they were all expressed to a higher degree in Huh7 compared to HepG2 cells, as was farnesoid X receptor (FXR; *NR1H4*). LXR is a lipogenic nuclear receptor, which promotes transcription of genes related to DNL and TAG uptake from lipoproteins; FXR, which primarily regulates bile acids and induces lipid disposal, also increases hepatic TAG uptake (Lopez‐Velazquez et al. 2012). It may be that the increased expression of LXR and FXR in Huh7 cells led to increased TAG uptake and storage, suggesting that Huh7 cells are more responsive to the effects of exogenous fat supply than HepG2 cells.

When cultured in FBS, the amount of DNL in both Huh7 and HepG2 cells could be considered supraphysiological; however, when cultured in HS DNL accounted for 18–25% in both cell lines, which is comparable to that reported for subjects with NAFLD (Donnelly et al. 2005; Lambert et al. 2014). The lower amount of DNL in cells cultured in HS may be due to the higher quantity of TAG in this serum: both SFA and PUFA are known inhibitors of the two major lipogenic transcription factors, SREBP‐1c and ChREBP (Dentin et al. 2005; Ye and DeBose‐Boyd 2011). In addition, we found that insulin signaling improved in both cell lines as a result of culture in HS, possibly due to FBS being rich in insulin and albumin (Gstraunthaler and Lindl 2011 2011), both of which can lead to insulin resistance. The relative insulin insensitivity in cells cultured in FBS compared to HS may have caused a reduction in gluconeogenesis; this would provide increased precursors for DNL, which remains upregulated in insulin resistance, thus reproducing the selective hepatic insulin resistance seen in vivo in humans (Schwarz et al. 2003). Taken together, our data indicates that culture of both cell lines in HS results in a more insulin‐sensitive phenotype and allows the role of insulin resistance in steatosis development to be investigated.

Both Huh7 and HepG2 cells have been found to secrete dense, lipid‐poor lipoproteins (Javitt 1990; Gibbons et al. 1994; Meex et al. 2011); culturing in HS improved lipoprotein profiles in both cell lines (Pramfalk et al. 2016; Steenbergen et al. 2013). Whether this results from a change in function due to HS‐cultured hepatocytes displaying a more differentiated phenotype, or simply an increase in FA availability for transfer to apoB‐containing particles, is not clear. It is well established that provision of exogenous FAs results in increased TAG content of lipoprotein particles secreted from HepG2 cells (Ellsworth et al. 1986; Dashti and Wolfbauer 1987; Wu et al. 1996). It is plausible that both the increase in secretion and oxidation in hepatoma cells in HS is a result of increased supply of FA. In this study, the only exogenous FA provided were that in the culturing sera. The low levels of essential FAs seems to have led to the elongation and desaturation of the product of DNL (C16:0): the most notable difference between FA composition of our cells and that seen in vivo, was an increase in palmitoleic (C16:1*n*‐7) and vaccenic acid (C18:1*n*‐7), and a reduction in linoleic (C18:2*n*‐6) and *α*‐linolenic acids (C18:3*n*‐3) and their products (Hodson et al. 2008; Arendt et al. 2015). This suggests the use of HS, as well as routine supply of exogenous FAs in the culture media, may assist in overcoming FA deficiency, thus lowering DNL and increasing TAG secretion and FA oxidation.

Culturing in HS altered the intracellular FA composition of the cells, resulting in a higher PUFA content. PUFAs were primarily abundant in the PL fraction of Huh7 cells, however, they were more abundant in the TAG fraction of HepG2 cells. HepG2 cells carry the I148M variant of the *PNPLA3* gene, which causes remodeling of hepatocellular LDs and results in increase PUFA and reduced stearate in intracellular TAG, which is in line with our results (Romeo et al. 2008; Peter et al. 2014; Ruhanen et al. 2014). In contrast, Huh7 cells have limited protein levels of PNPLA3, which results in enzyme activity equivalent to cells expressing the wild‐type protein (Ruhanen et al. 2014). Additionally, carriers of the *PNPLA3* I148M variant are suggested to have lower DNL‐derived intrahepatic fat and to secrete poorly‐lipidated apoB particles (Pirazzi et al. 2012; Sevastianova et al. 2012), which may explain the consistently lower rate of DNL and the tendency (in FBS but not HS) to secrete less TAG in HepG2 compared to Huh7 cells. The effect of this genotype on models investigating FA metabolism is, therefore, an important consideration.

Our study has some limitations. Firstly, we have focused primarily on cellular FA metabolism, specifically TAG and PL and the differences in FA supply between HS and FBS. Previous studies have shown that bile acid and cholesterol metabolism are improved in HepG2 cells with HS (Pramfalk et al. 2016), as well as hepatocyte morphology, cytochrome P450 enzyme function and differentiation (Pramfalk et al. 2016; Steenbergen et al. 2013). Our results demonstrate changes in metabolic function with limited changes in gene expression, especially in Huh7 cells. This led us to focus on differences in substrate supply between sera; however, the reduction in IGF‐1 signaling in Huh7 cells after culture in HS we observed may suggest a more differentiated phenotype in these cells. The sera were also used at different concentrations: HS at 2% and FBS 10%. We found cells were not viable when cultured with 2% FBS (data not shown), while previous work did not find any difference between culturing in 2% or 5% HS (Pramfalk et al. 2016). Given that the FA content of media was higher in 2% HS than 10% FBS, it is likely an increase in HS content would only have exaggerated the current findings. We measured media TAG as a surrogate measure of lipoprotein secretion. This means we may have missed differences in particle density, which has previously been shown to be lower in HepG2 compared to Huh7 cells when cultured in FBS (Meex et al. 2011). Finally, we have used two widely available and well‐characterized hepatocyte cell models; however, as development and validation of other models (e.g., iPSC and HepaRG) continues, investigation of the use of HS in these cells may lead to a FA‐metabolism model that more closely recapitulates a human hepatocyte phenotype.

In summary, we have shown that both HepG2 and Huh7 cells display improved insulin signaling and altered FA metabolism in response to culturing in HS; particularly, DNL was reduced and FAs appeared to be more readily mobilized for oxidation and secretion in both cell lines. Taken together, our data show that the type of serum that cells are cultured in along with the cell line utilized can influence intrahepatocellular FA metabolism. As a result, consideration of both of these parameters is required when developing a physiologically relevant, in vitro cellular model of FA metabolism for the study of the pathophysiology of NAFLD.

## Conflict of Interest

We confirm that this manuscript has not been presented or published elsewhere. P. J. Gunn, C. J. Green, C. Pramfalk and L. Hodson have no conflicts of interest to declare.
